# Experimental Infection of Mink with SARS-COV-2 Omicron Variant and Subsequent Clinical Disease 

**DOI:** 10.3201/eid2806.220328

**Published:** 2022-06

**Authors:** Jenni Virtanen, Kirsi Aaltonen, Kristel Kegler, Vinaya Venkat, Thanakorn Niamsap, Lauri Kareinen, Rasmus Malmgren, Olga Kivelä, Nina Atanasova, Pamela Österlund, Teemu Smura, Antti Sukura, Tomas Strandin, Lara Dutra, Olli Vapalahti, Heli Nordgren, Ravi Kant, Tarja Sironen

**Affiliations:** University of Helsinki, Helsinki, Finland (J. Virtanen, K. Aaltonen, K. Kegler, V. Venkat, T. Niamsap, L. Kareinen, R. Malmgren, O. Kivelä, N. Atanasova, T. Smura, A. Sukura, T. Strandin, L. Dutra, O. Vapalahti, H. Nordgren, R. Kant, T. Sironen);; Finnish Meteorological Institute, Helsinki (N. Atanasova);; Finnish Institute for Health and Welfare, Helsinki (P. Österlund);; Helsinki University Hospital, Helsinki (O. Vapalahti)

**Keywords:** COVID-19, SARS-CoV-2, mink, zoonoses, models, animals, coronavirus disease, severe acute respiratory syndrome coronavirus 2, viruses, respiratory infections, Finland

## Abstract

We report an experimental infection of American mink with SARS-CoV-2 Omicron variant and show that mink remain positive for viral RNA for days, experience clinical signs and histopathologic changes, and transmit the virus to uninfected recipients. Preparedness is crucial to avoid spread among mink and spillover to human populations.

SARS-CoV-2 has been detected in farmed and feral American mink (*Neovison vison*) in multiple countries, and extensive environmental contamination and human-to-mink and mink-to-human transmission has been documented (*1*–*5*). These factors have led to strict measures in mink farms and mink-farming countries to prevent the spread of the disease. In late 2021, a new SARS-CoV-2 variant (Omicron), characterized by possibly milder symptoms and more efficient human-to-human transmission, was detected, but its infectivity and spread in American mink is unknown (*6*,*7*).

We tested the response of American mink to the Omicron variant by infecting 3 male mink intranasally with 4 × 10^5^ plaque-forming units of the virus (Appendix). We conducted follow-up on infected mink for 7 days and performed histopathologic evaluation of upper and lower respiratory tracts on the last day of follow-up. We sampled saliva daily.

All experimentally infected mink showed mild to moderate signs of illness, including lethargy, anorexia, diarrhea, nasal and lacrimal discharge, and sneezing. Consistent with earlier experiments with other variants (*8*; D. Adney et al., unpub. data, https://www.biorxiv.org/content/10.1101/2022.01.20.477164v1), saliva samples tested PCR-positive 1 day postinfection (dpi) and remained that way throughout follow-up (Table; Appendix). Infectious virus was cultured 1–3 dpi. Even though some of the clinical signs could be caused by other factors, such as stress from the change of environment, their consistency with signs seen in studies of other variants, combined with PCR results, demonstrate that the Omicron variant also causes clinical disease in mink.

**Table:** Results of PCR and cell culture testing for SARS-CoV-2 Omicron variant in saliva samples from 3 experimentally infected mink and 2 uninfected recipient mink*

Mink ID	1 dpi	2 dpi	3 dpi	4 dpi	5 dpi	6 dpi	7 dpi	8 dpi	9 dpi	10 dpi
Infected mink										
451	+/ND	+/+	+/−	+/−	+/−	+/−	+/−			
453	+/(+)	+/−	+/−	+/−	+/−	+/−	+/ND			
455	+/−	+/−	+/(+)	+/−	+/−	+/−	+/−			
Recipient mink										
452	(+)/+	−/−	+/ND	+/−	+/−	+/+	+/−	+/−	+/−	(+)/−
454	-/+	−/+	(+)/−	+/−	+/−	+/ND	+/ND	+/−	+/−	+/−

*Plus sign alone indicates signal detected with both primers of Luna SARS-CoV-2 reverse transcription quantitative PCR Multiplex Assay Kit (New England BioLabs Inc., https://www.neb.com) and cycle threshold value from cell culture media was >5 cycles lower than that of the original saliva sample. Plus symbol in parentheses indicates signal detected only with 1 of 2 primers and cycle threshold value from cell culture media was 1–5 cycles lower than that of the original saliva sample. Minus sign indicates no signal with either of the primers and no cytopathic effect was detected in cell culture or cycle threshold value from the culture media was the same or higher than that of the original sample. dpi, days postinfection; ID, identification; ND, not done.

To study whether mink can transmit the virus, we placed 2 uninfected indirect contact mink in separate cages 10–20 cm from the cages of the infected mink and followed their progress for 10 days. Similar signs to the experimentally infected mink developed in both initially uninfected mink, and they were consistently PCR-positive from day 3 onward (Table), indicating mink-to-mink transmission. Infectious virus was detected in cell culture even before it was detected by PCR. Even though no evidence of mink-to-human transmission of the Omicron variant exists, it seems possible on the basis of our results and the information from studies of other variants.

Gross findings in the nasal cavity and lungs were subtle in both experimentally infected and recipient mink and consisted of hyperemia of respiratory mucosa with small amounts of viscous exudate and noncollapsed, dark-red, and wet pulmonary lobes. All mink showed histopathologic changes in the upper and lower respiratory tracts (Figure). We observed multifocal degeneration and loss of respiratory epithelium with variable mucosal and submucosal neutrophilic infiltration in the nose. The lumen contained sloughed epithelial cells, mucinous material, and degenerated neutrophils (Figure, panels A, C). Viral nucleoprotein was widely distributed beyond intact cells, within sloughed cells, and in mucosal respiratory epithelium (Figure, panels B, D). The olfactory epithelium was inconsistent and mildly affected with only focal viral antigen detection. Unlike in some experimental infections reported in rodents, clear pathology was observed in the lungs (*9*). In 2 inoculated and both recipient mink, pulmonary lesions (Figure, panels E, G) were associated with viral antigen expression (Figure, panels F, H) and characterized by multifocal to coalescing alveolar damage with degeneration or necrosis of alveolar septa, infrequent hyalin membrane formation, and variable proliferation of type II pneumocytes (Figure, panels I, J). Alveolar spaces contained macrophages, sloughed cells, edema, and hemorrhage. Bronchiolar epithelial degeneration and hyperplasia were variably present (Figure, panel K), and the lumen filled with few sloughed cells and neutrophils. Bronchi were lined by hyperplastic epithelium with increased numbers of goblet cells. Other consistent findings were vasculitis (Figure, panel L), perivasculitis, and perivascular and peribronchial edema. In 1 inoculated mink, we observed markedly thickened alveolar septa by mononuclear cells, marked proliferation of type II pneumocytes, intra-alveolar macrophages, few syncytial cells, bronchi and bronchiolar epithelial cell hyperplasia, vasculitis, and perivasculitis. We could not detect viral antigen in this mink. Strikingly, all evaluated mink lacked viral antigen in the epithelium of bronchi and bronchioles.

**Figure:**
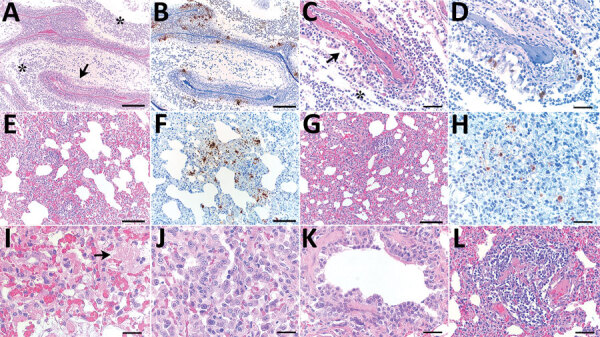
Histopathologic changes and SARS-CoV-2 expression in the upper and lower respiratory tracts of mink experimentally infected with Omicron variant at 7 days postinfection and recipient mink after 10 days of follow-up. A) Respiratory segment of the nose from an intranasally infected mink showing luminal accumulation of exudate (asterisks) and degeneration of mucosal epithelium (arrow) Scale bar indicates 500 µm. B) Viral antigen widely detected within nasal lumen and respiratory epithelium. Scale bar indicates 500 µm. C, D) Respiratory epithelium from a recipient mink depicting marked degeneration and loss (arrow in panel C) and intraluminal accumulation of sloughed cells and neutrophils (asterisk in pane C), and intraepithelial viral expression (panel D). Scale bars indicate 50 µm. E–H) Lungs from intranasally infected (E, F) and recipient (G, H) mink showing alveolar damage with intralesional presence of viral nucleoprotein. Scale bars indicate 200 µm in panels E–G and 50 µm in panel H. I, J) Marked degeneration and necrosis of alveolar septa and focal hyalin membrane (arrow in panel I) and prominent proliferation of type II pneumocytes (panel J) in an intranasally infected mink. Scale bars in indicate 25 µm). K, L) Recipient mink showing bronchiolar epithelial degeneration and hyperplasia (K) and vasculitis (L) with complete destruction of blood vessel wall and mononuclear cell infiltration. Scale bar indicates 50 µm in panel K and 100 µm in panel L. Hematoxylin and esosin stain and immunohistochemistry, hematoxylin counterstain.

The Omicron variant is different from other variants in its more efficient spread, primarily attributable to immune escape and likely milder symptoms in humans (*6*,*7*). These factors make preventing virus introduction into mink farms through asymptomatic humans more difficult, creating a more substantial risk for the formation of virus reservoirs among farmed or feral mink. This study shows that mink can be infected by Omicron and, crucially, efficiently transmit the virus to other mink. Despite the reports of lower virulence of Omicron, mink experience clinical disease and nasal and pulmonary microscopic lesions that closely resemble infection with previously reported variants in mink and humans (*8*). Clarifying the clinical signs will help detect the virus among mink earlier. Questions remain about the risks that the spread of this easily transmitted variant among mink would create for public health, including transmission to humans and emergence of mink-specific mutations, followed by their spillover to human population.

This article was preprinted at https://www.biorxiv.org/content/10.1101/2022.02.16.480524v2.

Appendix Additional information about experimental infection of mink with SARS-COV-2 Omicron variant and subsequent clinical disease
