# Supplementary material for: Experimental Infection of Mink with SARS-COV-2 Omicron Variant and Subsequent Clinical Disease
Source: Emerg Infect Dis. 2022 Jun;28(6):1286–8. doi: 10.3201/eid2806.220328 (PMC9155874; doi:10.3201/eid2806.220328)
Supplement: Appendix — Additional information about experimental infection of mink with SARS-COV-2 Omicron variant and subsequent clinical disease [file 22-0328-Techapp-s1.pdf]

Article DOI: https://doi.org/10.3201/eid2806.220328

Experimental Infection of Mink with SARS-COV-2 Omicron Variant and Subsequent Clinical Disease

Appendix

Materials and Methods

Virus Stock and Cell Line

Omicron variant (original patient sample: hCoV-19/Finland/THL-202126660/2021, EPI_ISL_8768822 (Gisaid)) of SARS-CoV-2 (10^6 PFU/ml) was acquired from the Finnish Institute of Health and Welfare. We ensured that the viral genome in the stock (OM393712) remained unchanged, including the furin cleavage site that mutates particularly rapidly in cell culture for Omicron, with a protocol described in (*1*). It has been reported that a few key mutations (e.g., the N501Y mutation) present in α, β, Gamma, and Omicron variants improve the affinity of the SARS-CoV-2 spike S1 protein to mouse ACE-2 and increase infectivity of SARS-CoV-2 in standard BALB/c mice (*1*).

TMPRSS2-expressing clone of Vero E6 cells (VE6T) (*2*) were grown in minimal essential eagle's medium (MEM, Sigma-Aldrich) including 10% (cell maintenance) or 2% (infection experiments) fetal bovine serum (FBS, ThermoFisher), 2 mM L-glutamine, 100 IU/mL penicillin, 100 µg/mL streptomycin, 0.205 µg/ml of amphotericin B (Fungizone, Thermo Scientific), and Voriconazole (Sigma-Aldrich, according to manufacturer's instructions).

Animals, Infection, and Euthanasia

A total of 5 male American mink (*Neovison vison*) were transported to the University of Helsinki biosafety level-3 (BSL-3) facility at the same time and acclimatized to custommade cages (located 10–20 cm from each other) with nest boxes for 3 days with ad libitum water and food. For the experimental infection, 3 of the mink were anesthetized with 30 µl of Ketaminol (100 mg/ml, Intervet) and Domitor (1 mg/ml, Orion Pharma) via i.m. administration and 3 were inoculated intranasally with 200 µL of virus stock into both nostrils and the remaining 2 with

phosphate-buffered saline (PBS). The animals were held in an upright position for a few seconds to allow the liquid to flush downwards in the nasal cavity. Revertor (5 mg/mL, Scanvet) was given (15uL) as an α2-adrenergic antagonist for reversing clinical effects of sedation. Two mink were left uninfected. All mink were monitored daily for signs of illness (changes in posture or behavior, rough coat, apathy, ataxia, runny nose, diarrhea etc.). At the end of the experiment, the mink were anesthetized with Ketaminol and Domitor (40 µl), sampled for blood, and euthanized in a CO2 chamber. Experimental procedures were approved by the Animal Experimental Board of Finland (license number ESAVI/33259).

Sample Collection

Saliva samples (oral swabs) were collected before the infection, and every day post infection with foam swabs (Virocult, MWE) into 200 µl PBS and culture media. Animals were necropsied immediately after euthanasia. Representative fresh samples from all lung lobes were collected and frozen for virological examinations. Lungs were then inflated with 10% neutral buffered formalin and fixed for 48 h. For histopathology, each lung lobe (right cranial, left cranial, right medial, right caudal and left caudal) were trimmed into three consecutive cross sections of ≈0.5 cm thick starting at the lobe hilum and moving toward the posterior of the lobe along the main branching bronchus. The head was separated from the carcass by disarticulation of the atlanto-occipital joint, the skull was removed exposing the brain and the entire was placed in 10% neutral buffered formalin. After 48 h fixation, the brain was removed and the head sawn longitudinally in the midline using a diamond saw. Slices of 0.3 cm were prepared from the nasal cavity including vestibular, respiratory and olfactory segments, and gently decalcified for 4 days in 14% EDTA solution (Tritrimex ® III, Nederland).

Histology and Immunohistochemistry

Trimmed lung lobes and decalcified nasal cavity sections were routinely processed, paraffin-wax embedded, cut at 3–5 µm and stained with hematoxylin–eosin (HE) or subjected to immunohistochemistry (IHC) for the detection of SARS-CoV-2 antigen. Briefly for IHC, slides were deparaffinized and incubated for 20 min at 99°C in 10 mM citrate buffer (pH 6) for heat-induced antigen retrieval. Endogenous peroxidase was performed by immersion in 3% hydrogen peroxide for 10 min. After incubation with 10% bovine serum albumin in phosphate-buffered saline, sections were incubated for 60 minutes at room temperature (RT) with rabbit polyclonal anti- SARS-CoV-2 nucleoprotein antibody diluted 1:3000 in animal-free blocker and diluent

solution (R.T.U. Animal-Free Blocker and Diluent; Vector Laboratories, Burlingame, Ca, USA). Polymer-linked to HRP (BrightVision + Poly-HRP kit; ImmunoLogic, Duiven, Netherlands) was used as secondary antibody incubating in a humid chamber for 30 min at RT. The reaction was visualized with right DAB Substrate kit (ImmunoLogic, Duiven, Netherlands) and slight counterstain with Harris hematoxylin. As negative control, the primary antibody was substituted with rabbit IgG Isotype control (1:1000).

PCR

RNA was extracted from saliva samples collected in PBS with Viral RNA Minikit (Qiagen) according to manufacturer's instructions. PCR tests were performed with Luna SARS-CoV-2 RT-qPCR Multiplex Assay Kit (NEB). Samples that gave signal with both primers were considered positive (+) and samples that only gave signal with one primer weak positive ((+)).

Virus Culture

Saliva samples collected in culture media were subjected to cell culture in 6-well plates by adding the whole sample and 2 ml of culture media to the cells. Cells were grown at 37°C for 9 days or until cytopathic effect (CPE) was detected. If CPE was detected, a 140 µl sample from the culture media was taken and RNA was extracted with QIAamp 96 Virus QIAcube HT kit (Qiagen, off-board lysis) and tested with PCR as described above. Culturing result was considered positive if Ct values from the culture media were clearly lower (more than 5 cycles) than that of the original saliva sample (+). If Ct values were 1–5 cycles lower, culturing results was considered a possible positive ((+)). If Ct values were similar or bigger than those of the original sample, result was considered negative.

References

1. Kant R, Kareinen L, Smura T, Freitag TL, Jha SK, Alitalo K, et al. Common Laboratory Mice Are Susceptible to Infection with the SARS-CoV-2 Beta Variant. Viruses. 2021;13:2263. PubMed https://doi.org/10.3390/v13112263

2. Rusanen J, Kareinen L, Szirovicza L, Uğurlu H, Levanov L, Jääskeläinen A, et al. A Generic, Scalable, and Rapid Time-Resolved Förster Resonance Energy Transfer-Based Assay for Antigen Detection-SARS-CoV-2 as a Proof of Concept. MBio. 2021;12:e00902-21. PubMed https://doi.org/10.1128/mBio.00902-21

Appendix Table 1. Cycle threshold values from saliva samples in PCR with Luna SARS-CoV-2 RT-qPCR Multiplex Assay Kit (2019-nCoV_N1/2019-nCoV_N2)*

	Infected mink			Recipient mink	
dpi	451	453	455	452	454
1	28.15/28.27	23.27/23.85	25.58/25.66	no ct/36.67	no ct/no ct
2	28.23/28.55	34.63/34.17	29.66/30.16	no ct/no ct	no ct/no ct
3	14.15/13.86	14.9/14.61	15.85/15.71	33.36/33.38	38.68/no ct
4	25.3/26.27	21.19/21.99	25.66/26.72	28.71/30.06	31.13/32.27
5	32.44/33.22	25.87/26.00	23.71/23.83	34.13/34.12	26.46/26.59
6	27.49/28.01	29.79/30.77	32.17/33.97	30.19/30.75	33.57/34.16
7	25.03/26.91	27.07/28.78	27.82/29.80	30.91/33.51	27.25/29.12
8	N/A	N/A	N/A	28.07/28.29	31.81/32.01
9	N/A	N/A	N/A	30.69/32.56	34.7/36.36
10	N/A	N/A	N/A	37.41/no ct	32.79/34.05

*ct, cycle threshold; dpi, days post infection; N/A, not applicable.

Appendix Table 2. Cycle threshold values from culture medias of saliva samples in PCR with Luna SARS-CoV-2 RT-qPCR Multiplex Assay Kit (2019-nCoV_N1/2019-nCoV_N2) at the time cytopathic effect was observed*

	Infected mink			Recipient mink	
dpi	451	453	455	452	454
1	ND	20.40/22.54	No CPE	19.97/20.78	31.28/32.74
2	17.26/18.26	30.09/35.12	No CPE	no ct/no ct	35.75/41.25
3	13.78/14.30	14.78/15.38	13.48/14.08	ND	no ct/no ct
4	No CPE	No CPE	No CPE	No CPE	No CPE
5	no ct/no ct	no ct/no ct	30.05/32.48	no ct/no ct	28.32/30.50
6	31.03/31.99	no ct/no ct	ND	23.04/25.04	34.73/39.09
7	No CPE	ND	No CPE	No CPE	ND
8	N/A	N/A	N/A	33.82/39.09	no ct/no ct
9	N/A	N/A	N/A	No CPE	No CPE
10	N/A	N/A	N/A	no ct/no ct	No CPE

*CPE, cytopathic effect; ct, cycle threshold; ND, not done.
